# Kevlar^®^, Nomex^®^, and VAR Modification by Small Organic Molecules Anchoring: Transfusing Antibacterial Properties and Improving Water Repellency

**DOI:** 10.3390/molecules28145465

**Published:** 2023-07-17

**Authors:** Efrosyni Frousiou, Efstathios Tonis, Georgios Rotas, Anna Pantelia, Savvas G. Chalkidis, Nikolaos S. Heliopoulos, Antonia Kagkoura, Dionysios Siamidis, Angeliki Galeou, Anastasia Prombona, Kostas Stamatakis, Nikos Boukos, Georgios C. Vougioukalakis

**Affiliations:** 1 Laboratory of Organic Chemistry, Department of Chemistry, National and Kapodistrian University of Athens, 15771 Athens, Greece; 2 Laboratory of Organic Chemistry, Department of Chemistry, University of Ioannina, 45110 Ioannina, Greece; 3 700 Military Factory, Supreme Military Support Command, 50 Anapafseos, 18648 Piraeus, Greece; 4 Theoretical and Physical Chemistry Institute, National Hellenic Research Foundation, 48 Vassileos Constantinou Avenue, 11635 Athens, Greece; 5 Siamidis S.A., Industrial Zone, Inofita, 32011 Viotia, Greece; 6 Institute of Biosciences and Applications, National Centre for Scientific Research “Demokritos”, Patriarchou Grigoriou E’ & Neapoleos Str., 15341 Agia Paraskevi Attica, Greece; 7 Institute of Nanoscience and Nanotechnology, National Centre for Scientific Research “Demokritos”, Patriarchou Grigoriou E’ & Neapoleos Str., 15341 Agia Paraskevi Attica, Greece

**Keywords:** Kevlar^®^, Nomex^®^, viscose, surface modification, graft polymerization, water repellency, antibacterial activity

## Abstract

The surface modification of fabrics composed of Kevlar^®^, Nomex^®^, or VAR was extensively investigated. Kevlar^®^ and Nomex^®^ are widely-utilized aramid materials, whereas VAR is a technical fabric comprising 64% viscose, 24% para-aramid (Kevlar^®^), 10% polyamide, and 2% antistatic fibers. Both aramid materials and cellulose/viscose exhibit exceptional mechanical properties that render them valuable in a wide range of applications. For the herein studied modification of Kevlar^®^, Nomex^®^, and VAR, we used small organic molecules 3-allyl-5,5-dimethylhydantoin (ADMH) and 3-(acrylamidopropyl)trimethylammonium chloride (APTAC), which were anchored onto the materials under study via graft polymerization. By doing so, excellent antibacterial properties were induced in the three studied fabrics. Their water repellency was improved in most cases as well. Extensive characterization studies were conducted to probe the properties of the modified materials, employing Raman and FTIR spectroscopies, Scanning Electron Microscopy (SEM), and thermogravimetric analysis (TGA).

## 1. Introduction

The use of body armor is highly effective in reducing fatalities in military environments and law enforcement, protecting against bullet penetration and wounds from knives and spikes [1]. However, personal armor usually burdens military and law enforcement personnel with additional weight and difficulty in movement, which can lead to unintended consequences, such as premature exhaustion and limited ability to move in orrespond appropriately to life-threatening situations. For this reason, it is essential to find a balance among survivability, mobility, and flexibility [2].

The above-mentioned requirements are very often met by materials characterized as technical fabrics. In particular, woven panels made of *p*-aramid fibers (e.g., Kevlar^®^ and Twaron^®^) have the ability to absorb kinetic energy and are used as armor materials [3], while *m*-aramid fibers (Nomex^®^) are used to impart flame-resistance properties that do not degrade with washing and wear. Additionally, mixed fabrics of aramid and viscose fibers, such as VAR, are used to increase the comfort and flexibility of the finished product [4].

Aramids are a class of robust synthetic materials consisting of long-chain aromatic polyamide fibers. Their outstanding strength, compared to other synthetic fabrics, is attributed to the fact that their chain molecules are highly oriented along the fiber axis. Thus, a higher portion of the chemical bond contributes to the fiber’s strength [5]. Nomex^®^ (*m*-aramid) and Kevlar^®^ (*p*-aramid) are examples of high-performance materials that possess superior mechanical properties, fire resistance, excellent thermal, chemical, and oxidative stabilities, poor solubility in many solvents, as well as increased tensile strength [6,7,8,9]. Fabrics manufactured from these high-performance fibers find wide use in many demanding applications and are worn by firemen, military and medical personnel, race car drivers, and more [10]. VAR is another example of a technical fabric, mainly consisting of viscose (made from natural sources of regenerated cellulose), para-aramid fibers, and antistatic materials, altogether contributing to the fabric’s exceptional flame resistance [11,12,13]. It is worth mentioning that cellulose offers the potential for chemical modification via radical graft polymerization, utilizing initiators like peroxides and hydroperoxides as initiators [14].

On a different note, 3-allyl-5,5-dimethylhydantoin (ADMH) is a hydantoin derivative containing a N-H group that can endow outstanding antimicrobial properties upon N–H to N–Cl bond conversion [15]. By “depositing” a chlorine atom in the N-X covalent bond, long-term chemical stability, high shelf life, and reproducibility of the disinfecting properties are ensured [16]. ADMH has been effectively grafted onto a wide range of materials, like high-performance fibers, including Kevlar^®^ and Nomex^®^, polymers- and cellulose-based surfaces, etc., granting them substantial biocidal activities [6,7,8,14,17,18]. 3-(Acrylamidopropyl)trimethylammonium chloride (APTAC) is a chloride anion-containing ammonium compound, which has also been used for grafting onto different natural polymers [19].The incorporation of APTAC into the structure of different materials, like gels, composite fillers polysaccharides, and cotton fibers, has endowed valuable properties, like considerable antimicrobial effects on bacteria, selective heavy metal adsorption for the removal of silver and arsenic waste, and temperature responsiveness to stimuli [19,20,21,22,23,24,25]. Drug delivery with controlled substance release has also been successfully implemented [26].

Polymeric materials can be modified in a tailor-designed manner to meet specific requirements, thus leading to enhanced functionality, strength, water repellency, antibacterial protection, and more [27,28]. Moreover, compounds possessing antibacterial and water-repellent properties have a crucial role in various fields. For example, the risk of infections can be greatly decreased by inhibiting the growth of bacteria [29,30]. Water repellency is equally significant, especially concerning textiles and coatings, as it prevents water damage, enhances durability, and improves the longevity of products [31].

In our present work, we aim to improve the properties of Kevlar^®^ intended for anti-ballistic plates and fabrics, as well as Nomex^®^ and VAR, used as carrier fabrics for atomic shielding. We employ the organic compound ADMH, which has been anchored onto a variety of materials and fabrics before [5,14,17,18], as well as the commercially available compound APTAC. Our main goal is to introduce anti-bacterial activity and increase the water-repellency of all three materials. To achieve this, we anchor the two organic molecules onto the fabrics, using a straightforward protocol, and assess the success of this modification by a range of state-of-the-art characterization techniques: Raman and FTIR spectroscopies; Scanning Electron Microscopy (SEM); and thermogravimetric analysis (TGA). We then investigate the impact of our surface modification protocol on the wettability of the materials and examine the acquisition of anti-bacterial properties. As mentioned above, ADMH has been successfully utilized for the modification of Kevlar^®^, Nomex^®^, and cellulose fibers before [6,7,8,14]. However, we decided to employ it herein, given that: (i) it has not been utilized with VAR; and (ii) to use it as a comparison/benchmark to the use of APTAC, which has not been utilized for this reason in the past. Equally important, we herein show that APTAC, as well as ADMH, can induce an increase in fabrics’ water repellency in most cases, in contrast to previously published protocols using ADMH [32,33], and also visualize ADMH’s successful anchoring onto aramid fibers via SEM for the first time.

## 2. Materials and Methods

### 2.1. Materials

Chemicals were obtained from commercial sources: Scharlau (benzoyl peroxide), Sigma Aldrich (poly(ethylene glycol)diacrylate, allyl bromide,), Fluorochem (5,5-dimethylhydantoin), Tokyo Chemical Industries (3-(acrylamidopropyl)trimethylammonium chloride), Fischer Chemical (dimethylformamide), Lach:ner (acetone), Chemlab (potassium persulfate), and used without any further purification. For the purpose of our study, we used three technical fabrics with inherent heat- and flame-resistant properties, engineered for professional clothes on a semi-industrial scale, provided by Siamidis S.A., Oinofyta, Greece. The first fabric, colored yellow, consisted of 100% Kevlar^®^ fibers, with a dry weight of 200 g∙m^−2^, 1/1 plane, 600 Dtex yarn count, 9.5 ends∙cm^−1^ density of warp and weft. The second (Nomex^®^), colored blue, had a weight of 220 g∙m^−2^, 2/1 plane, Nm 55/2 warp and weft yarn number, fabric count of 29 wrap threads∙cm^−1^ and 26 weft threads∙cm^−1^, and consisted of 98% Nomex^®^ fibers (93% *m*-aramid/5% *p*-aramid fibers), as well as 2% P140 antistatic fibers. The third (VAR), colored grey, had a weight of 185 g∙m^−2^, 2/1 plane, fabric count of 24 wrap treads/10 cm, and 24 weft threads/10 cm, consisting of viscose fibers, para-aramid, polyamide, and antistatic fibers with the following composition: 64% viscose/24% para-aramid (Kevlar^®^)/10% polyamide/2% antistatic fibers.

The *Escherichia coli (E. coli)* strain used was DH5α purchased from Invitrogen. The medium used for growing and maintaining the bacterial liquid cultures was Luria–Bertani (LB) growth medium (1.0% Tryptone (Panreac), 0.5% yeast extract (Merck), 1.0% sodium chloride (Panreac), pH adjusted to 7.3±0.1 with 5.0 N NaOH (Merck)). The unicellular cyanobacterium *Synechococcus* sp. PCC7942 was obtained from the Collection Nationale de Cultures de Microorganisms CNCM, Institut Pasteur, Paris, France.

### 2.2. Modification Methods

**Figure 1:**
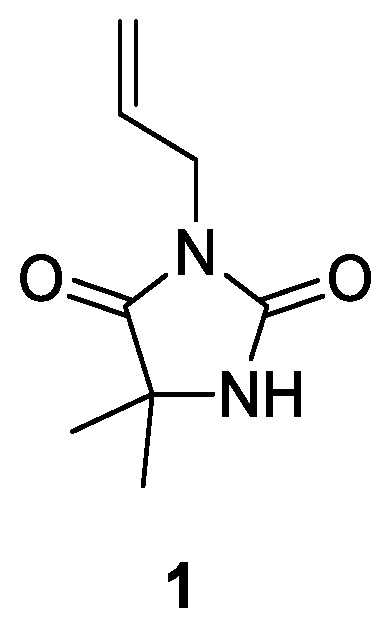
Chemical structure of 3-allyl-5,5-dimethylhydantoin (ADMH) **1**.

Synthesis of **ADMH (1**, see Figure 1 below**) [34]**

In a round flask equipped with a magnetic stirrer, a solution of 5,5-dimethylhydantoin (15.6 mmol, 1 equiv) was added to acetone, followed by the addition of anhydrous potassium carbonate (46.8 mmol, 3 equiv). The mixture was refluxed for 30 min. Then, allyl bromide (16.8 mmol, 1.08 equiv) was added, and the reflux was continued for 19 h. The solvent was then removed under reduced pressure. The reaction mixture was extracted with ethyl acetate (3 × 50 mL), the organic phase was dried over sodium sulfate, and filtered, and the solvent was removed under reduced pressure. The final product was purified by column chromatography using an elution system of petroleum ether/ethyl acetate: 1/1. Yield: 49%. ^1^H-NMR (200 MHz, DMSO-*d*_6_) *δ*8.32 (s, 1H), 5.95–5.61 (m, 1H), 5.21–4.85 (m, 2H), 3.93 (d, *J* = 7.0 Hz, 2H), 1.28 (t, *J* = 6.2 Hz, 6H). ^13^C-NMR (101 MHz, DMSO-*d*_6_) *δ* 177.5, 155.4, 132.9, 116.3, 58.3, 39.1, 25.2.

Anchoring of ADMH onto Kevlar^®^ (**KEV2**) [7,18]

Benzoyl peroxide (BPO, 500mg, 2mmol) and poly(ethylene glycol) diacrylate (PEG-dia) (0.8 mL, 1.5 mmol) were added to a bath of H_2_O. ADMH (1g) was dissolved in the solvent and added into the bath dropwise. A piece of Kevlar^®^ fabric (8 cm diameter, 700 mg) was immersed in the bath twice. Afterward, the fabric was placed in the oven at 50 °C for 5 min and cured for 1 h at 130 °C. Next, the fabric was washed with water and was left to dry in the oven at 60 °C for 24 h.

Chlorination procedure of **KEV3 [7,18]**

The fabric was placed in a 50:1 commercial aqueous sodium hypochlorite solution (NaOCl) at room temperature for 30 min under stirring. Then, it was washed with water and dried in the oven at 60 °C for 24 h.

Anchoring of ADMH onto Nomex^®^ **(NOM2**)

The experimental procedure followed for the modification of the Nomex^®^ fabric with ADMH (**NOM2**) was identical to the procedure followed for **KEV2** (vide supra).

Chlorination procedure of **NOM3**

The experimental procedure followed for the chlorination of **NOM2** was identical to the procedure followed for **KEV3** (vide supra).

**Figure 2:**
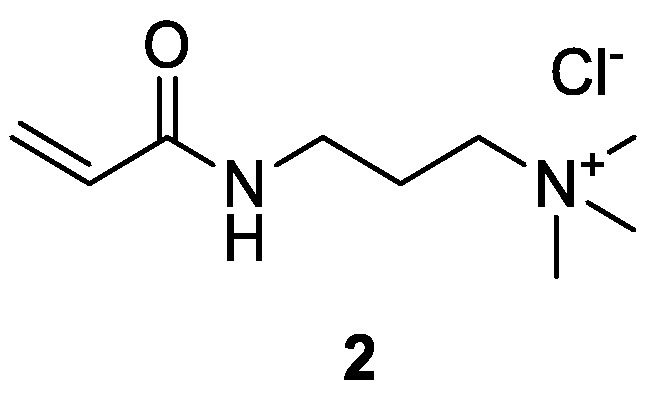
Chemical structure of 3−(acrylamidopropyl)trimethylammonium chloride (APTAC) **2**.

Anchoring of APTAC onto KEVLAR^®^ **(KEV1**, see Figure 2 below**)**

The experimental procedure followed for the modification of **KEV1** was identical to the procedure followed for **KEV2** (vide supra).

Anchoring of APTAC onto Nomex^®^(**NOM1**)

The experimental procedure followed for the modification of Nomex with APTAC (**NOM1**) was identical to the procedure followed for **KEV2** (vide supra).

Anchoring of APTAC onto VAR **(VAR1**)

APTAC (3 mL) was dissolved in DMF (50 mL) and stirred at room temperature for 15 min. Next, the VAR fabric (8 cm diameter, 800mg) was immersed in the bath. Potassium persulfate (1.2 g, 4.4 mmol) was then added, and the mixture was stirred for 7 h at 70 °C. Finally, the fabric was washed with water and dried in the oven at 60 °C for 24 h.

### 2.3. Characterization Methods of Modified Fabrics

Infrared (FTIR) spectra were obtained on a Fourier Transform IR spectrometer (Equinox 55 from Bruker Optics) equipped with a single reflection diamond ATR accessory (DuraSamp1IR II by SensIR Technologies). Raman measurements were recorded with a Renishaw confocal spectrometer upon excitation at 514 nm at 10% of 0.265 mW power and with ×L50 length. The corresponding data were obtained and analyzed with Renishaw Wire and Origin software. Thermogravimetric analyses were acquired using a TGA Q500 V20.2 Build 27 instrument by TA in a nitrogen (purity >99.999%) inert atmosphere. Scanning Electron Microscopy (SEM) secondary electron images were acquired utilizing an FEI Quanta Inspect microscope operating at 10 kV accelerating voltage.

Wettability

The wettability of the fabric samples was determined by the water contact angle (WCA) test to investigate the hydrophilicity or hydrophobicity of the fabrics. The measurements were done using a custom-made experimental setup, with distilled water in the static mode of the sessile droplet method, on raw and treated samples. Using a 2.0MP 500× USB digital microscope, images were taken of a 5 μL water droplet on the surface of each substrate within 30 s of droplet deposition. Droplet profiles were analyzed using standard MATLAB functions. Briefly, images were first converted to grayscale. For the calculation of WCA, the intersection between the apparent vertical symmetry axis of the drop and the solid/liquid interface was chosen as the origin of the coordinates. The edge profiles were treated in polar coordinates (r) since polynomial functions can be fitted satisfactorily to the smoothly varying r(𝜃) with 𝜃. The WCA values were converted back to Cartesian coordinates, resulting from the derivatives at the contacts of the left and right sides of the droplet with the surface. The value of WCA for the specific sessile droplet was obtained as the average of the right and left contact angles, the deviation of which was less than 2°. Four sessile droplets were placed in each sample, and the final WCA value was obtained from the average of the four measurements. The WCAs were measured at room temperature.

Antibacterial activity

The bacterial protection of the modified samples was determined according to two methods: one providing qualitative and one providing quantitative results for the antibacterial action of the substances that modified the fabrics. In the first method, the Gram-negative bacterium *E. coli* DH5α was used. A single colony of DH5α was inoculated into LB nutrient broth and grown overnight at 37 °C with constant agitation at 220 rpm. After this, 1 mL of a 1–5 × 10^8^ CFU/mL working culture was added, with vigorous shaking to evenly distribute the bacteria, to 150 mL of LB nutrient-agar. The bacterial culture was used to inoculate the upper agar layer, consisting of 5 ± 0.1 mL LB nutrient with 7.5 g/L agar (precooled to 45 ± 1 °C), on the surface of which 25 ± 5 mm-diameter test specimens were placed in a petri dish. The bottom layer consisted of LB culture medium containing 12% agar free of bacteria. Using sterile forceps, the samples were pressed onto the upper agar layer until the texture of the fabric was uniformly imprinted. Petri dishes were incubated for 18–24 h at 37 °C. The level of antibacterial activity was assessed by examining the extent of bacterial growth in the contact zone between the agar and the specimen and, if present, the extent of the inhibition zone around the specimen.

An in situ antimicrobial susceptibility test (AST) method (method two) based on measurements of cyanobacteria chlorophyll *α* (Chl *α*) fluorescence was used to quantify the antibacterial protection of the modified fabrics [35,36]. In summary, the unicellular cyanobacteria *Synechococcus* sp. PCC7942 (Collection Nationale de Cultures de Microorganismes CNCM, Institut Pasteur, Paris, France) were cultured in the BG11 medium [37] under white, fluorescent light (100 μE·m^−2^·s^−1^) in an orbital incubator (Galenkamp INR-400) at 31 °C and aeration with 5% *v*/*v* CO_2_ in air [38]. To determine the antibacterial protection of fabrics, an appropriate number of PCC7942 cells was harvested from the culture suspensions by centrifugation (5000 rpm, 5 min). It was resuspended in buffered BG11, so that the concentration of Chl *a* was at 52.0 μg∙mL^−1^. Chl *α* concentration was determined in *N*,*N*-dimethylformamide (DMF) extracts of cell pellets according to Moran (1982) [39]. A drop of the cyanobacteria sample, volume 0.05 mL, was transferred to each fabric sample, creating a spot up to 3.0 mm in diameter. The cyanobacterial cell proliferation rate in VAR samples was monitored by measuring Chl *α* fluorescence every 24 h for ten days, using a PEA fluorometer (PEA, Hansatech Instruments Ltd., Norfolk, UK), after first adjusting the samples to darkness for 15 min with an appropriate clip. *F_0_* was the first reliable Chl *a* fluorescence measurement at 20 μs. According to Equation (1) and the *M*_i_ index, the normalized fluorescence changes of the cyanobacteria in each sample were calculated.

(1) $$M_{\iota }=\frac{F_{0_{i}}-F_{0_{0}}}{F_{0_{0}}}\times \;100$$

where $F_{0_{0}}$ is the value of Chl *α* fluorescence of cyanobacterium at zero contact time and $F_{0_{i}}$ is the value of Chl *α* fluorescence of cyanobacterium after 1,2, …,*i* days.

The material’s antibacterial action was represented by the Bacterial Protection Index (BPI)$\Pi_{i}$, given by Equation (2):

(2) $$\Pi_{i}=\frac{M_{U_{i}}-M_{T_{i}}}{M_{U_{i}}}\times 100$$

where $M_{U_{i}}$, is the change in cyanobacterial Chl *αF_0_* value on the untreated sample after the days of incubation and $M_{T_{i}}$ is the change in cyanobacterial Chl *αF*_0_ value on the treated sample after the days of incubation [40,41,42].

Throughout the measurements, the samples were kept in an incubator under white, fluorescent light (100 µE∙m^−2^∙s^−1^) at 31 °C. All experiments were performed in three replicates using different cyanobacterial cell cultures.

## 3. Discussion

**Figure 3:**
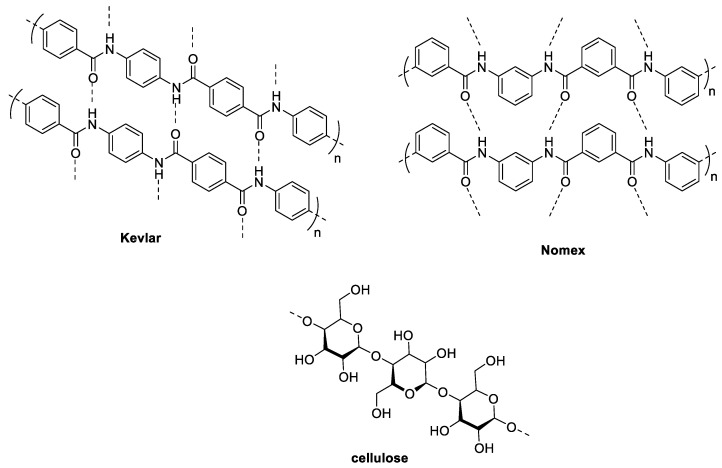
Chemical structure of Kevlar^®^, Nomex^®^, and cellulose.

**Scheme 1:**
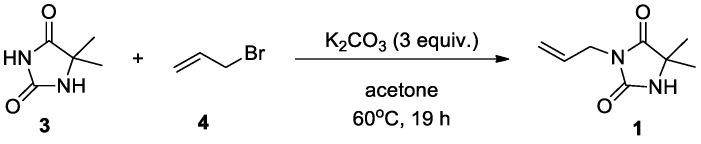
Synthesis of ADMH **1**.

As mentioned above, the objective of the present study was to thoroughly investigate the surface modification of Kevlar^®^, Nomex^®^, and VAR materials (Figure 3), using 3-allyl-5,5-dimethylhydantoin (ADMH, **1**) and 3-(acrylamidopropyl)trimethylammonium chloride (APTAC, **2**). Nomex^®^ (*m*-aramid) fabrics are used in situations where the garments need to provide heat and flame protection, while VAR and Kevlar^®^ (*p*-aramid) fabrics are ideal for the carrier and woven panels of anti-ballistic vests, respectively. Although ADMH has been previously utilized to provide antibacterial properties to fabrics, including Kevlar^®^, Nomex^®^, and cellulose [5,14,17,18], we decided to study its use as a benchmark compound, along with the use of commercially available APTAC, which has not been utilized in the past. Moreover, neither ADMH nor APTAC has been used on VAR. Following a published procedure on a similar compound, we began with the synthesis of ADMH, by reacting 5,5-dimethylhydantoin with allyl bromide [34]. The reaction was initially carried out with methanol as the solvent, using potassium hydroxide as the base, at 60 °C. However, the reaction yield was relatively low. After switching to potassium carbonate as the base and acetone as the solvent, under reflux conditions (Scheme 1), the reaction yield increased to 49%.

We then began investigating the conditions for anchoring ADMH and APTAC onto Kevlar^®^, Nomex^®^, and VAR, employing a radical-initiated graft polymerization approach. The polymerization conditions can vary, among others, by using different solvents and initiators (e.g., water-soluble or not) [43]. Our purpose was to develop an efficient and straightforward protocol leading to the robust anchoring of APTAC and ADMH onto high-performance fabrics in the presence of PEG-dia, which wasutilized as a copolymer. Anchoring onto the fabrics took place via the radical homopolymerization of the vinyl moieties of the monomers with benzoyl peroxide (BPO) as the initiator [7]. In the literature, there are alternative procedures for the anchoring of compounds onto fabrics, such as the cure-pad-dry process [9].

**Scheme 2:**
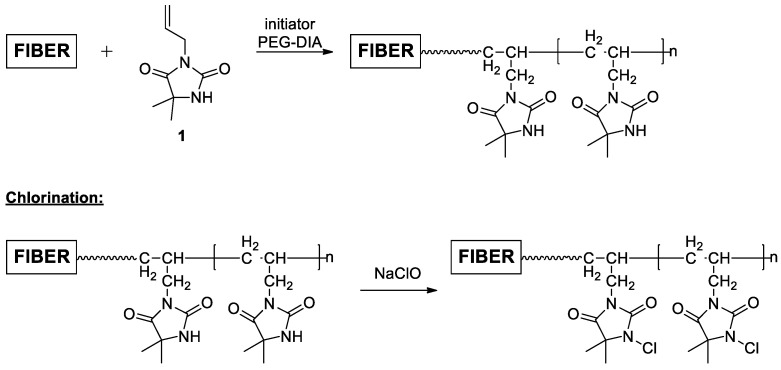
ADMH grafting polymerization onto the fibers, followed by the chlorination procedure [6,7,8,9,18].

ADMH and APTAC were attached to the materials by following the same experimental procedure [7]. Scheme 2 shows schematically the mode of ADMH binding to the fabrics. Following the polymer formation on the surface of the fiber (binding), chlorination via treatment with sodium hypochlorite took place in order to install the N-Cl bonds, thus improving the modified fabrics’ antibacterial properties. In the case of anchoring APTAC on VAR, PEG-dia was not utilized, while potassium persulfate was used as the radical initiator instead of BPO. An organic solvent was used for anchoring APTAC onto VAR, which is a disadvantage of the method for future application in a production process. On the contrary, the use of aqueous solutions for anchoring ADMH and APTAC onto fabrics composed of aramid fibers was a comparative advantage of the process.

Characterization and morphology of the Kevlar^®^, Nomex^®^, and VAR surfaces

**Figure 4:**
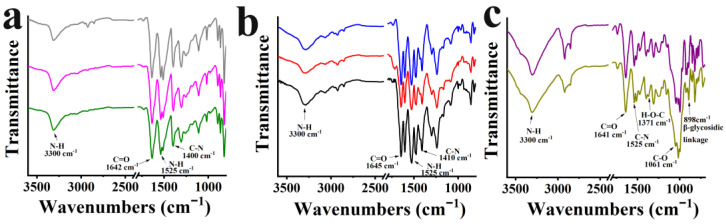
FTIR spectra for (**a**) Kevlar^®^ fabric with APTAC, **KEV1** (green), Kevlar^®^ fabric with ADMH, **KEV2** (pink), and untreated Kevlar^®^ fabric (grey); (**b**) Nomex^®^ fabric with APTAC, **NOM1** (blue), Nomex^®^ fabric with ADMH, **NOM2** (red), and untreated Nomex^®^ fabric (black); and (**c**) VAR fabric with APTAC, **VAR1** (purple), and untreated VAR fabric (yellow).

Kevlar^®^ and Nomex^®^ textiles have distinct infrared (FTIR) spectra characterized by strong absorption bands. In the case of Kevlar^®^, its FTIR spectra displayed prominent bands associated with carbonyl amide stretching at 1642 cm^−1^, C-N stretching at 1400 cm^−1^, and N-H vibrational modes at 1525 and 3300 cm^−1^ (Figure 4a) [44]. These characteristic bands were clearly visible in the FTIR spectra of modified Kevlar^®^ fabrics **KEV1** and **KEV2**. Similarly, the modified Nomex^®^ fabric exhibited bands at 1645, 1410, 1525, and 3300 cm^−1^, which corresponded to the stretching vibrations of the C=O, C-N, and N-H moieties, respectively, that were also observed in **NOM1** and **NOM2** (Figure 4b) [45]. No new distinct bands were observed in the spectra of modified Kevlar^®^ and Nomex^®^ materials, as the carbonyl amide stretching of the anchored compounds was masked by the strong bands existing at the same wavenumbers in untreated fibers. VAR fabric, comprising Kevlar^®^ and viscose fibers, exhibited a wide range of prominent FTIR bands. These included a strong band at 1641 cm^−1^, attributed to the stretching vibration of the carbonyl amide, bands at 1390 and 1525 cm^−1^, associated with the stretching of the C-N bonds, N-H vibrational modes at 3300 cm^−1^, bands at 1371, 1479, and 2918 cm^−1^, arising from the C-H stretching, and a band at 1371 cm^−1^, originating from the bending of H-O-C in the polysaccharide rings of viscose (Figure 4c) [44,46]. Moreover, the characteristic band at 898 cm^−1^ indicated the presence of β-glycosidic linkages between glucose units, while the band at 1061 cm^−1^ corresponded to the vibration of C-O in the pyranose ring, which forms part of the viscose chain backbone [47]. As before, those bands were evident in **VAR1**, masking the expected carbonyl vibrations of the anchored compounds (Figure 4c).

**Figure 5:**
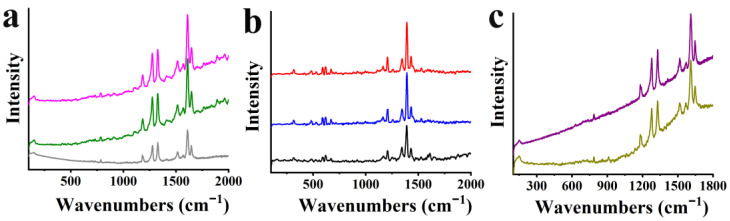
Raman spectra (514 nm) for (**a**) Kevlar^®^ fabric with APTAC, **KEV1** (green), Kevlar^®^ fabric with ADMH, **KEV2** (pink), and untreated Kevlar^®^ fabric (grey); (**b**) Nomex^®^ fabric with APTAC, **NOM1** (blue), Nomex^®^ fabric with ADMH, **NOM2** (red), and untreated Nomex^®^ fabric (black); and (**c**) VAR fabric with APTAC, **VAR1** (purple) and untreated VAR fabric (yellow).

The Raman spectra of both reference and modified fabrics were recorded upon excitation at 514 nm. The Raman spectra of Kevlar^®^ contain numerous, well-defined bands between 100 and 1800 cm^−1^, displaying a variety of features [48]. Among the most prominent bands are those observed at 1614 and 1649 cm^−1^, which correspond to the stretching vibrations of the C-C phenyl ring and the C=O group, respectively [49]. These bands were clearly visible in Figure 5a. Following surface modification, the Raman spectra of the modified Kevlar^®^ fabrics **KEV1** and **KEV2** exhibited minimal differences compared to the reference spectra, as depicted in Figure 5a. A similar trend was observed for the Nomex^®^-based material, where the strong bands observed in the unmodified fabric (Figure 5b) also dominated the spectra of the modified Nomex^®^ fabrics, **NOM1** and **NOM2** (Figure 5b). Similar observations were made for the VAR-modified material, **VAR1**, as the prominent bands associated with Kevlar^®^were clearly visible [49].

**Figure 6:**
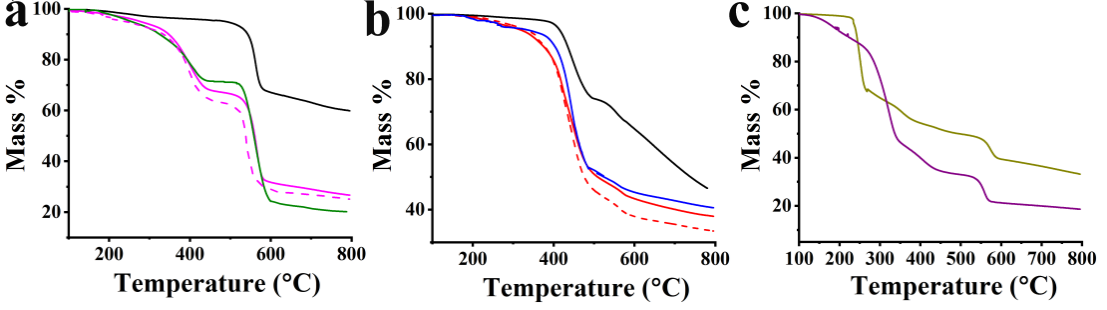
Thermogravimetric analysis graphs obtained under nitrogen atmosphere for (**a**) Kevlar^®^ fabric with APTAC, **KEV1** (green), Kevlar^®^ fabric with ADMH, **KEV2** (pink), Kevlar^®^ fabric with ADMH + Cl, **KEV3** (dashed pink), and untreated Kevlar^®^ fabric (grey); (**b**) Nomex^®^ fabric with APTAC, **NOM1** (blue), Nomex^®^ fabric with ADMH, **NOM2** (red), Nomex^®^ fabric with ADMH + Cl, **NOM3** (dashed red), and untreated Nomex^®^ fabric (black); and (**c**) VAR fabric with APTAC, **VAR1** (purple), and untreated VAR fabric (yellow).

Thermographs were also utilized to monitor the modification processes taking place on the functionalized materials. The thermogravimetric analysis (TGA) profile of the original Kevlar^®^ fabric aligned with previous findings, displaying a mass reduction of around 31% between 200 and 580 °C (Figure 6a) [50]. In contrast, the modified Kevlar^®^ fabrics, **KEV1** and **KEV2**, demonstrated a higher mass loss of 68% and 66% within the same temperature range, respectively. This increased mass loss can be attributed to the thermal degradation of the organic molecules grafted onto the fabrics. Additionally, fully functionalized fabric (after chlorination) **KEV3** demonstrated a 2% higher mass loss up to 580 °C compared to **KEV2**. The mass loss for modified Kevlar^®^ materials at higher temperatures suggests that the process of covalent modification leads to increased degradation of the polymer chains within the fabric. Simultaneously, the functionalized Nomex^®^ fabric **NOM2** and fully functionalized fabric (after chlorination) **NOM3** demonstrated an earlier onset of mass loss, starting at approximately 198 °C, in contrast to unmodified Nomex^®^ fabric (Figure 6b). Moreover, **NOM1** and **NOM2** exhibited significantly higher overall mass loss of 54%, 56% up to 580 °C, respectively. This increase in mass loss is attributed to the thermal decomposition of the covalently attached APTAC and ADMC molecules, respectively, indicating the successful modification of the Nomex^®^ fabric. In contrast, partially functionalized **NOM2**, which underwent similar treatment as **NOM3**, but lacked chlorination, exhibited a lower mass loss (56%) up to 580 °C compared to fully functionalized **NOM3** (61%), providing evidence of the successful chlorination treatment. Finally, the thermal stability of VAR was maintained up to 200 °C, with a subsequent mass loss of 54% up to 585 °C (Figure 6c). Conversely, **VAR1** displayed a higher mass loss of 78% within the same temperature range. These increased mass losses, observed for VAR-modified materials, serve as strong evidence for the successful functionalization and grafting of the organic components onto the fabric.

Scanning Electron Microscopy

**Figure 7:**
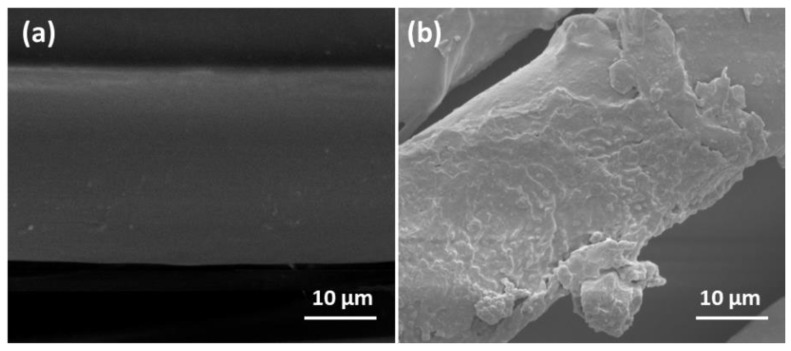
SEM images of untreated Nomex^®^ (**a**) and functionalized with ADMH Nomex^®^ (**NOM2**) (**b**).

The surface morphology of untreated and modified-with-ADMH (**NOM2**) Nomex^®^ materials was thoroughly studied by Scanning Electron Microscopy (SEM). As shown in Figure 7a, untreated Nomex^®^ fibers had a smooth texture with very few impurities. On the other hand, Figure 7b shows that the surface of the modified fiber was efficiently covered by the ADMH/PEG-DIA copolymer. Importantly, the Nomex^®^ fibers remained intact in **NOM2**, showing no indication of degradation. To the best of our knowledge, this is the first time that SEM imaging has been utilized for a protocol regarding the ADMH anchoring to aramid fibers. Figure 7b proves the successful modification of the Nomex^®^ material and explains the excellent results regarding its excellent antibacterial activity and water repellency (vide infra).

Wettability

**Figure 8:**
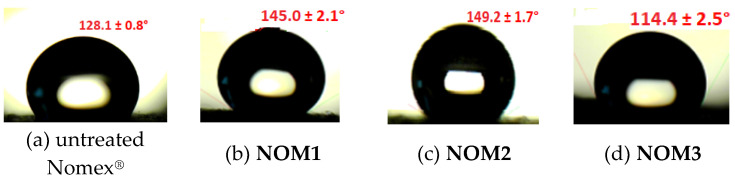
Wettability effect of Nomex^®^ fabrics. (**a**) Untreated Nomex^®^ fabric, (**b**) Nomex^®^ fabric modified with APTAC (**NOM1**), (**c**) Nomex^®^ fabric modified with ADMH (**NOM2**), and (**d**) Nomex^®^ fabric modified with ADMH + Cl (**NOM3**).

**Figure 9:**
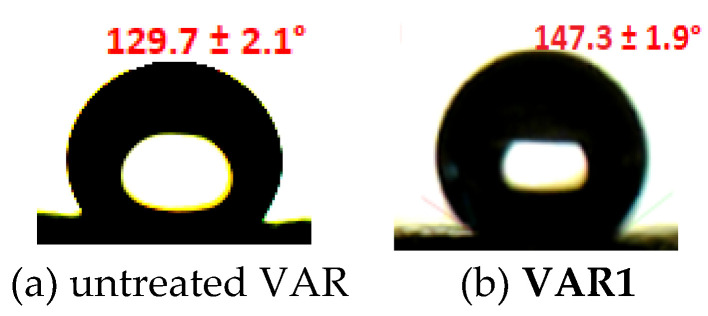
Wettability effect of VAR fabrics. (**a**) Untreated VAR fabric and (**b**) VAR fabric modified with APTAC (**VAR1**).

The value of the contact angle between a water droplet and the surface of a certain material/surface provides a straightforward evaluation of its wettability properties. When the value of the water contact angle is <90°, the fabrics are characterized as hydrophilic, while when the water contact angle has a value between 90 and 150°, they are characterized as hydrophobic [51]. According to our studies on the wettability of the investigated fabrics (Figure 8 and Figure 9), untreated Nomex^®^ and VAR fibers were characterized as hydrophobic, given that the water contact angle (WCA) value was greater than 90° (128.1° and 129.7°, respectively), while Kevlar^®^, with a WCA value of 0°, was characterized as hydrophilic.

Modification of Kevlar^®^ with ADMH or APTAC did not change the hydrophilicity of the untreated fabric, given that after 5 s, the water droplet was completely absorbed. On the contrary, Figure 8 and Figure 9, showing the wettability test results for the modified Nomex^®^ and VAR fabrics with APTAC, suggest an increase in the hydrophobicity of both materials. More specifically, the WCA value for the Nomex^®^ fabric increased from 128.1° to 145.0° (**NOM1**), while for the VAR fabric, it increased from 129.7° to 147.3° (**VAR1**). The modification of the Nomex^®^ fabric with ADMH also increased the WCA value to 149.2° (**NOM2**). Modification with ADMH + Cl slightly decreased the WCA value to 114.4° (**NOM3**) due to the hydrophilic groups. Nevertheless, the hydrophobicity of the fabric remained at an acceptable level. This is an interesting result which has not been reported in previous related studies [32,33].

Antibacterial properties

The evaluation of the antibacterial activity of modified fabrics was done by the agar plate diffusion test with the Gram-negative non-pathogenic bacteria *E. coli*. The fewer bacterial colonies growing under the sample, the greater the ability of the antibacterial substance to protect against bacteria. Table 1, Table 2 and Table 3 show the results for fabrics composed of Kevlar^®^, Nomex^®^, and VAR materials, respectively. The images of the samples after the completion of the test with the growth of the cells can be seen in the first row of the tables, while in the second row, we show the result of the test without the sample fabrics. Evaluation of these results showed that all three untreated materials did not inhibit bacterial growth. Fabrics modified with APTAC (**ΚEV1**, **NOM1**, **VAR1**) had good antibacterial activity without completely inhibiting the growth of bacteria (bacteriostatic activity), while fabrics treated with ADMH (**ΚEV2**, **NOM2**) and ADMH + Cl (**ΚEV3**, **NOM3**) completely inhibited the growth of bacteria and formed an inhibition zone. These results show a very good antibacterial effect of the substances (bactericidal activity).

**Figure 10:**
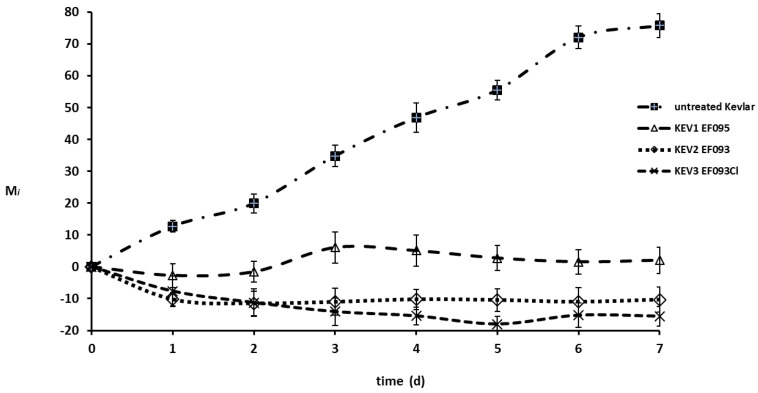
*M*_i_ evolution curves of cyanobacteria on Kevlar^®^ samples: (▪) untreated Kevlar^®^ fabric, (Δ) Kevlar^®^ fabric modified with APTAC (**KEV1**), (◊) Kevlar^®^ fabric modified with ADMH (**KEV2**), and (x) Kevlar^®^ fabric modified with ADMH + Cl (**KEV3**).

**Figure 11:**
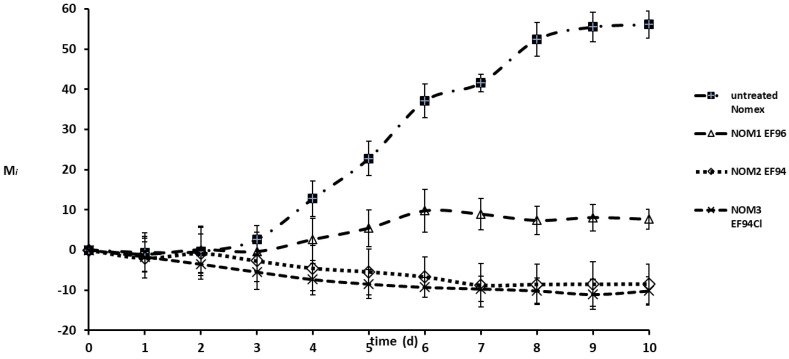
*M*_i_ evolution curves of cyanobacteria on Nomex^®^ samples: (▪) untreated Nomex^®^ fabric, (Δ) Nomex^®^ fabric modified with APTAC (**NOM1**), (◊) Nomex^®^ fabric modified with ADMH (**NOM2**), and (x) Nomex^®^ fabric modified with ADMH + Cl (**NOM3**).

**Figure 12:**
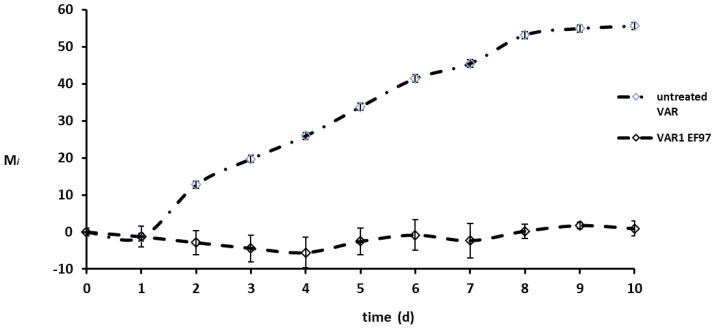
*M*_i_ evolution curves of cyanobacteria on VAR samples: (▪) untreated VAR fabric and (Δ) VAR fabric modified with APTAC (**VAR1**).

Figure 10, Figure 11 and Figure 12 show the quantification of the bacterial protection of the modified fabrics as a function of the change in the population of cyanobacteria, reflected in the changes in the *M*_i_ values. An increase in the population corresponds to an increase in the measured Chl *α* fluorescence intensity (*F_0_*) of the cyanobacteria, while a decrease in Chl *α* fluorescence intensity means that cellular degradation has begun, and the cells are dying. Thus, negative or lower values on comparable surfaces are interpreted as enhanced antibacterial behavior. The untreated samples of the three different fabrics showed an increase in *F_0_* values until the cyanobacteria growth cycle wascomplete on them, indicating that they didnot provide any bacterial protection. The fabrics modified with APTAC (**KEV1**, **NOM1**, **VAR1**) showed a small increase in the population of cyanobacteria, confirming the very good bacterial protection they provide, as shown by the results of the first method, while the substances ADMH and ADMH + Cl which modified **ΚEV2**, **NOM2**, and **ΚEV3**, **NOM3** fabrics, respectively, led to negative values of the change in the population of bacteria, indicating that they not only completely inhibit their growth but also kill them, imparting excellent antibacterial properties to the modified fabrics.

Table 4 shows the values of the Bacterial Protection Index (*Π*_i_) of the modified fabrics, resulting from the calculations using equation 2, after seven days of growth of cyanobacteria for the fabrics composed of Kevlar^®^ and ten days for the fabrics composed of Nomex^®^ and VAR. In relation to the above, the bacterial protection level of APTAC fabrics was very high, especially for **KEV1** (*Π*_7_ = 97.3) and **VAR1** (*Π*_10_ = 98.3), while the modification with ADMH and ADMH + Cl imparted excellent antibacterial properties to the modified fabrics composed of Kevlar^®^ and Nomex^®^ materials, with the corresponding Bacterial Protection Index having values above 100, which corresponds to total bacterial protection.

The excellent bacterial protection of Kevlar^®^, Nomex^®^, and VAR fabrics, when modified with ADMH or ADMH + Cl (N-halamine), is in agreement with other studies employing ADMH or ADMH + Cl on materials and fabrics composed of nylon, polyester, acrylic, polypropylene, or natural fibers [6,8]. ADMH is a hydantoin derivative containing an N-H group endowing antimicrobial properties. The antibacterial action of such compounds against Gram-negative (*E. coli*) and Gram-positive bacteria most probably originates from their penetration through bacterial membranes and the subsequent damage of various cytoplasmic components, like DNA and proteins [52]. Along these lines, N-halamines are generally among the most effective antimicrobials against a wide range of microorganisms because when the oxidizing halogen is depleted, after inactivating the microorganisms, it gets “recharged”, thus continuing to inactivate pathogens [53].

On the other hand, APTAC is a quaternary ammonium salt. Quaternary ammonium salts, after coating onto fabrics and other materials surfaces, can kill microorganisms through ionic interactions by attacking the cytoplasmic membranes, thus resulting in leakage of intracellular components. Nevertheless, they have relatively lower antibacterial efficacy compared with N-halamine compounds [54]. The herein-found antibacterial activity of APTAC on *E. coli* is in good agreement with the findings of another study [55]. In a nutshell, APTAC can penetrate the membranes acting as an antibacterial agent, albeit less efficiently than ADMH and ADMH + Cl.

## 4. Conclusions

Herein, we extensively investigated the surface modification of Kevlar^®^ and Nomex^®^ aramid fabrics, as well as VAR technical fabric, which are particularly useful in body armor (*p*-aramid materials), fire- and flame-retardant textiles (*m*-aramid materials), and in a wide range of applications in automotive, medical devices, and electronics industries. To modify the properties of these materials, we utilized 3-allyl-5,5-dimethylhydantoin (ADMH) and 3-(acrylamidopropyl)trimethylammonium chloride (APTAC). Both organic molecules tested were successfully anchored onto Kevlar^®^, Nomex^®^, and VAR fabrics via graft polymerization, using PEG-dia as the copolymer and BPO as the radical initiator. To thoroughly investigate the modified materials, characterization studies were conducted using Raman and FTIR spectroscopies, SEM imaging, and thermogravimetric analysis. Additionally, the wettability/water repellency of the fabrics was evaluated, as well as their antibacterial properties. Nomex^®^ and VAR fabrics showed enhanced water repellency when modified with ADMH, while the modification of Nomex^®^ with APTAC resulted in a slight decrease in hydrophobicity, albeit maintaining its hydrophobic character. On the other hand, modified Kevlar^®^ fabrics did not lose their hydrophilic character. The antibacterial properties of all modified fabrics were at an excellent level. Optimum antibacterial and water-repellent results were obtained with ADMH on Nomex.

## Figures and Tables

**Table 1:** Antibacterial effect of Kevlar^®^ fabrics. (**a**) Untreated Kevlar^®^ fabric, (**b**) Kevlar^®^ fabric modified with APTAC (**KEV1**), (**c**) Kevlar^®^ fabric modified with ADMH (**KEV2**), and (**d**) Kevlar^®^ fabric modified with ADMH + Cl (**KEV3**).

	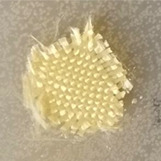	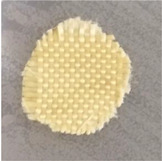	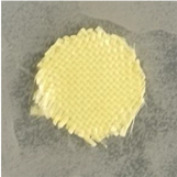	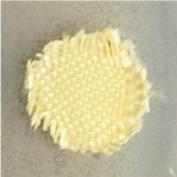
	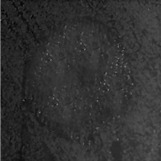	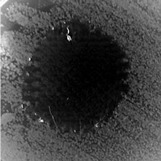	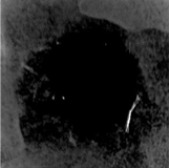	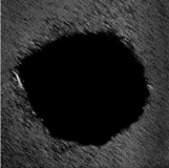
Growth	heavy	none/slight	none	none
Assessment	-	good effect/ limit of efficacy	good effect	good effect
	(a) Untreated Kevlar^®^	(b) **KEV1**	(c) **KEV2**	(d) **KEV3**

**Table 2:** Antibacterial effect of Nomex^®^ fabrics. (**a**) Untreated Nomex^®^ fabric, (**b**) Nomex^®^ fabric modified with APTAC (**NOM1**), (**c**) Nomex^®^ fabric modified with ADMH (**NOM2**), and (**d**) Nomex^®^ fabric modified with ADMH + Cl (**NOM3**).

	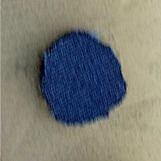	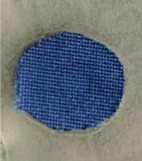	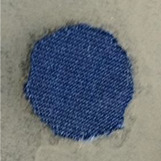	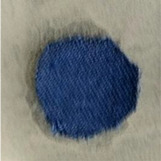
	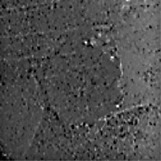	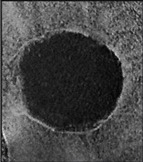	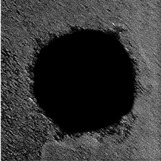	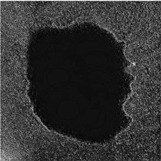
Growth	heavy	slight	none	none
Assessment	-	limit of efficacy	good effect	good effect
	(a) Untreated Nomex^®^	(b) NOM1	(c) NOM2	(d) NOM3

**Table 3:** Antibacterial effect of VAR fabrics. (**a**) Untreated VAR fabric and (**b**) VAR fabric modified with APTAC (**VAR1**).

	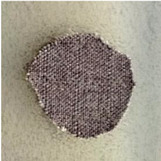	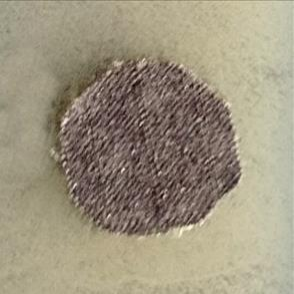
	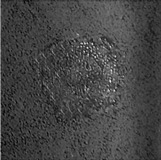	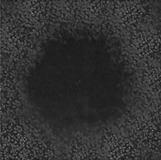
Growth	heavy	none/slight
Assessment	-	good effect/ limit of efficacy
	(a) Untreated VAR	(b) **VAR1**

**Table 4:** *Π*_i_ (Bacterial Protection Index) values for the examined Kevlar^®^, Nomex^®^, and VAR samples. Kevlar^®^ (**KEV1**), Nomex^®^ (**NOM1**) and VAR (**VAR1**) fabric modified with APTAC; Kevlar^®^ (**KEV2**) and Nomex^®^ (**NOM2**) fabric modified with ADMH; Kevlar^®^ (**KEV3**) and Nomex^®^ (**NOM3**) fabric modified with ADMH + Cl.

	untreated Kevlar^®^	**KEV1**	**KEV2**	**KEV3**
*Π*_7_	0.0	97.3	113.6	120.5
	untreated Nomex^®^	**NOM1**	**NOM2**	**NOM3**
*Π*_10_	0.0	86.3	115.1	118.1
	untreated VAR	**VAR1**		
*Π*_10_	0.0	98.3		

## Data Availability

Data will be made available on request.

## Sample Availability

Samples of the compounds and the fabrics are available from the authors.
